# Clinical and laboratory features distinguishing between *Deinagkistrodon acutus* and *Daboia siamensis* envenomation

**DOI:** 10.1186/s40409-018-0179-2

**Published:** 2018-12-27

**Authors:** Hung-Yuan Su, Shih-Wei Huang, Yan-Chiao Mao, Ming-Wen Liu, Kuo-Hsin Lee, Pei-Fang Lai, Ming-Jen Tsai

**Affiliations:** 1 Department of Emergency Medicine, E-Da Hospital and I-Shou University, Kaohsiung, Taiwan; 2 The School of Chinese Medicine for Post Baccalaureate, I-Shou University, Kaohsiung, Taiwan; 3 Department of Emergency Medicine, Buddhist Tzu Chi General Hospital, Hualien, Taiwan; 4 Division of Clinical Toxicology, Department of Emergency Medicine, Taichung Veterans General Hospital, Taichung, Taiwan; 5 Department of Emergency Medicine, Ditmanson Medical Foundation Chia-Yi Christian Hospital, No. 539, Zhongxiao Road, East District, Chiayi City, 600 Taiwan

**Keywords:** Coagulopathy, *Deinagkistrodon acutus*, *Daboia siamensis*, Snakebite, Thrombocytopenia

## Abstract

**Background:**

There are 6 species of venomous snakes in Taiwan. Two of them, *Deinagkistrodon acutus* (*D. acutus*) and *Daboia siamensis* (*D. siamensis*), can cause significant coagulopathy. However, a significant proportion of patients with snakebites cannot identify the correct snake species after envenomation, which hampers the application of antivenom. Hence, the differential diagnosis between the two snakebites by clinical presentations is important. This study aims to compare their clinical and laboratory features for the purpose of differential diagnosis between the two snakebites.

**Methods:**

We retrospectively reviewed the medical records of patients who arrived at the emergency department due to *D. acutus* or *D. siamensis* envenomation, between 2003 and 2016, in one medical center in eastern Taiwan. Since these snakebites are rare, we also included 3 cases reported from another hospital in central Taiwan.

**Results:**

In total, 15 patients bitten by *D. acutus* and 12 patients by *D. siamensis* were analyzed. Hemorrhagic bulla formation and the need for surgical intervention only presented for *D. acutus* envenomation cases (Both 53.3% vs. 0.0%, *P* = 0.003). As to laboratory features, lower platelet counts (20.0 ×  10^3^/μL [interquartile range, 14–66 × 10^3^/μL] vs. 149.0 × 10^3^/μL [102.3–274.3 × 10^3^/μL], *P* = 0.001), lower D-dimer level (1423.4 μg/L [713.4–4212.3 μg/L] vs. 12,500.0 μg/L [2351.4–200,000 μg/L], *P* = 0.008), higher proportion of patients with moderate-to-severe thrombocytopenia (platelet count < 100 × 10^3^/μL) (80% vs. 16.7%, odds ratio (OR) = 20.0, 95% CI, 2.77–144.31; *P* = 0.002), and lower proportion of patients with extremely high D-dimer (> 5000 ng/mL) (16.7% vs. 66.7%, adjusted OR = 0.1 (95% CI, 0.01–0.69; *P* = 0.036) were found among cases of *D. acutus* envenomation compared to *D. siamensis* envenomation. The combination of hemorrhagic bulla, thrombocytopenia, and a lack of extremely high D-dimer had good discriminatory power (area under the curve (AUC) = 0.965; 95% CI, 0.904–1.00) for distinguishing *D. acutus* from *D. siamensis* envenomation.

**Conclusions:**

The presentation of moderate to severe thrombocytopenia (platelet count < 100 × 10^3^/μL) and hemorrhagic bulla formation may indicate *D. acutus* envenomation. However, the envenomed patient with extremely high D-dimer levels may indicate a *D. siamensis* envenomation. These findings may help diagnose and select the right antivenom in patients with unknown snakebites who present significant coagulopathy.

## Background

Snake envenomation is a serious and important public health issue worldwide, including in Taiwan [1, 2]. Taiwan is a natural habitat for more than 40 snake species, including 6 types of venomous snakes with clinical importance, namely: *Protobothrops mucrosquamatus* (Taiwan habu), *Trimeresurus stejnegeri* (Taiwan bamboo viper)*, Deinagkistrodon acutus (D. acutus), Daboia siamensis (D. siamensis)*, *Bungarus multicinctus* (banded krait), and *Naja atra* (Taiwan cobra) [3, 4]. Among the abovementioned venomous species, the first 4 belong to the Viperidae family, which possess hemotoxic venom that can cause varying degrees of bleeding tendency in humans. In general, most patients with Taiwan habu or Taiwan bamboo viper envenomation present with local hemotoxic effects; however, the manifestation of systemic coagulopathy is rare and mild [5]. A previous study by Chen et al. had reported only 6% of Taiwan habu and 0% of Taiwan bamboo viper envenomation presenting coagulopathy and less than 1% of Taiwan habu presenting severe coagulopathy [6]. However, in *D. acutus and D. siamensis* envenomation, systemic coagulopathies including thrombocytopenia, prolonged prothrombin time (PT), activated partial thromboplastin time (APTT), fibrinogen consumption and D-dimer production are common [7–9].

*D. acutus*, also called the hundred pacer, is the largest snake of the crotalinae subfamily in Taiwan [4]. This species can inject a large amount of venom at each envenomation, with the venom containing several hemotoxins including pro-coagulation proteins, such as thrombin-like enzyme (TLE), as well as anticoagulation proteins, such as factor IX/X inhibitor and platelet aggregation inhibitor [10–13]. *D. siamensis*, which belongs to the viperinae subfamily, has venom composed of mixed hemotoxins, including pro-coagulation proteins, such as factor V, IX, and X activator, protease inhibitors, and phospholipase A2 [13–15]. The main habitats of *D. acutus* and *D. siamensis* are very similar and both are distributed from the eastern to southern regions of Taiwan [3]. Victims of these two snakebite types are rare and only account for 2.4% (*D. acutus*) and 2.9% *(D. siamensis*) of the total venomous snakebites in eastern Taiwan [16]. In addition to Taiwan, these two snakes are also concurrently distributed in other Southeastern Asian countries, such as Laos and Vietnam and southern China [17].

Currently, the definitive treatment for these types of snakebites is horse-derived antivenom, specific for *D. acutus* and *D. siamensis*. However, previous studies have shown that about 30% of patients with venomous snakebites were unable to identify the correct snake species [3, 6]. This leads to difficulty in administering the correct antivenom, especially in those patients with significant coagulopathy. Although the concurrent use of two specific antivenoms may be practiced clinically, the high cost in generating antivenom, low inventory, and its side effects, such as serum sickness, should also be considered [18–21]. The correct clinical differential diagnosis between these two types of snakebites is paramount.

Unlike four other types of venomous snakebites were well investigated in Taiwan, there is still a lack of data to distinguish clinical features between *D. acutus* and *D. siamensis* envenomation. The aim of this study was to investigate the clinical and laboratory differences between *D. acutus* and *D. siamensis* envenomation, which can help emergency physicians make correct clinical diagnosis, especially in those patients with systemic coagulopathy but unknown snake envenomation.

## Methods

### Study population

We conducted a retrospective study of patient data on *D. acutus* and *D. siamensis* envenomation, who were admitted to the Hualien Tzu Chi Medical Center, the only medical center in eastern Taiwan, between 2003 and 2016. The patient data collection methodology was previously described [5, 16]. Briefly, patient medical records were collected for those admitted with snakebites, using the computerized chart system and International Classification of Diseases, 9th Revision, Clinical Modification codes 989.5, E905.0, E905.9, E906.2, and E906.5. For *D. acutus* and *D. siamensis* envenomation, classification of the snake species was based on patient identification from a photograph taken by cell phone, or bringing the snake to the emergency department (ED). We only included patients with venomous snakebites who received specific antivenoms for *D. acutus* or *D. siamensis* and excluded those without receiving antivenom for suspicion of dry bite. Patients who could not confirm the correct snake species and who received more than one type of antivenom were also excluded. Three authors independently reviewed the clinical records of the included patients to confirm that each patient had a relevant history, typical manifestation, and consistent antivenom administration.

Due to the rare incidence of *D. acutus* and *D. siamensis* envenomation, we also searched case reports of *D. acutus* or *D. siamensis* envenomation in Taiwan registered during the most recent 10 years in the literature. However, only Cheng et al. had published 3 cases of *D. acutus* envenomation from Taichung Veterans General Hospital in 2017 [7]. After contacting the author, we obtained the de-identifiable patients’ original data and included the 3 patients in the study.

### Demographic data and definition of variables

Patients’ age, gender, site of snakebite, comorbidities, envenomation details, clinical presentation, laboratory results, treatment, initial antivenom therapy timing, and total antivenom dose were analyzed. The laboratory analysis included initial patient data obtained upon arriving at the ED, including hematology, biochemistry, and coagulation profiles. We defined leukocytosis as a white blood cell count (WBC) of > 11.0 × 10^3^/μL; moderate-to-severe thrombocytopenia as a platelet count of < 100 × 10^3^/μL [22]; non-coagulation in prothrombin time (PT) and activated partial thromboplastin time (aPTT) as either PT or aPTT beyond laboratory upper limits; fibrinogen consumption as fibrinogen levels of < 1.0 g/L; extremely high D-dimer levels > 5000 ng/mL [23]; acute renal impairment as creatinine levels > 1.4 mg/dL [8]; and venom-induced consumption coagulopathy as a disseminated intravascular coagulation (DIC) score ≥ 5 points [24, 25]. All the reference standards had been checked for consistency during the study period. If the laboratory value was beyond the laboratory upper or lower limit, it was recorded as the upper or lower limit, respectively. If the patient’s initial laboratory tests were not performed in the ED, this was recorded as a missing value in the database. All patient records and information were de-identified and anonymized before the analysis. The institutional review board of the Hualien Tzu Chi Medical Center approved the study protocol (IRB106–128-B).

### Statistical analyses

The normality of the quantitative variables’ distribution was tested by the Kolmogorov-Smirnov test (*P* > 0.10). Comparison of continuous variables between the two types of snakebites was conducted using the Mann-Whitney U-test or Student’s *t* test, depending on variable distribution. The chi-square test or Fisher’s exact test was applied for categorical variables as appropriate. Normally distributed data are expressed as mean ± standard deviation (SD), while non-parametric data were expressed as the median [25th–75th interquartile range]. All statistical tests were two-tailed while a *P* value of < 0.05 was considered statistically significant. Odds ratios (ORs) were calculated using logistic regression analysis. In addition, receiver operating characteristic (ROC) curves for different combination of the significant variables were calculated to determine which clinical manifestations can distinguish these two types of snakebite. All data were analyzed through the software SPSS, version 12.0 (IBM Corp.; Armonk, NY, USA).

## Results

Respective totals of 15 and 12 patients with *D. acutus* and *D. siamensis* envenomation were analyzed. Among them, 2 patients of each envenomation type identified the species by bringing the snake to the ED; the remaining patients identified the species by a photograph taken on a cell phone or by examining the standard pictures of Taiwanese venomous snakes provided by the Centers for Disease Control, R.O.C. (Taiwan).

### Demographic, clinical, and laboratory features

Comparisons of patient demographic, clinical, and laboratory characteristics are listed in Tables 1 and 2. As to clinical features, there was no significant difference between the two types of snakebite in age, gender, bite location or elapsed time until hospital arrival (Table 1). Eight of 15 patients (53.3%) with *D. acutus* envenomation received surgical intervention (debridement, fasciotomy, or skin graft) due to suspicion of compartment syndrome by clinical symptoms (4 patients), or tissue infection or necrosis according to local findings (4 patients), but no *D. siamensis* envenomation patient underwent surgery (*P* = 0.003; Table 1). Among the 8 surgical patients with *D. acutus* envenomation, mixed types of bacteria were detected in the surgical wounds of 5 patients (62.5%). *Morganella morganii* and *enterococcus faecalis* were the leading isolated pathogens (Table 3). In relation to local signs, hemorrhagic bulla formation was presented by more than half of the *D. acutus* envenomation patients, but by none of those with *D. siamensis* envenomation (53.3% vs. 0.0%, P = 0.003; Table 1).

**Table 1:** Comparison of clinical and laboratory characteristics between patients with *Deinagkistrodon acutus* and *Daboia siamensis* envenomation

	*Deinagkistrodon acutus* (*n* = 15)	*Daboia siamensis* (*n* = 12)	P value
Age	47.7 ± 13.81	55.0 ± 10.06	0.140
Male gender	14 (93.3)	9 (75.0)	0.294
Season			
Summer & fall	14 (93.3)	6 (50.0)	0.024
Winter and spring	1 (6.7)	6 (50.0)	
Bite area			
Upper limbs	10 (66.7)	10 (83.3)	0.408
Lower limbs	5 (33.3)	2 (16.7)	
Operation	8 (53.3)	0 (0)	0.003
Local signs			
Swelling	15 (100)	12 (100)	1.000
Ecchymosis	9 (60)	4 (33.3)	0.252
Hemorrhagic bulla	8 (53.3)	0 (0)	0.003
Time to arrive hospital (hr)	2.0 (1–7.5)	7.5 (1–12)	0.373
Total dose of antivenom (vial)	6.0 (4–10)	4.0 (4–6)	0.067
Duration of hospitalization (day)	5.0 (2–27)	3.0 (2–7)	0.183
ED laboratory data:			
WBC (× 10^3^/μL)	10.3 ± 2.98	12.1 ± 3.90	0.187
Hb (g/dL)	14.1 ± 2.77	14.0 ± 1.39	0.889
PLT (× 10^3^/μL)	20.0 (14–66)	149.0 (102.3–274.3)	0.001
PT (sec)	100.0 (100–100)	75.0 (11–100)	0.025
aPTT (sec)	150.0 (25–191) (*n* = 14)	29.0 (25–160)	0.118
Fibrinogen (g/L)	0.5 (0.25–0.93) (*n* = 13)	0.5 (0.25–0.95)	0.689
D-dimer (μg/L)	1423.4 (713.4–4212.3) (n = 12)	12,500.0 (2351.4–200,000.0)	0.008
DIC score	7.0 (5–7)	5.5 (4–6)	0.041
AST (IU/L)	29.0 (22–35)	36.0 (24–101) (*n* = 11)	0.330
ALT (IU/L)	22.0 (18.0–44.5) (n = 13)	25.0 (20–38)	0.852
BUN (mg/dL)	14.0 (10.8–19.5) (n = 14)	17.0 (12.5–30.8)	0.193
CRE (mg/dL)	0.9 (0.78–1.33) (n = 14)	1.2 (0.93–2.38)	0.118
CK (IU/L)	242.0 (213.5–748.0) (n = 13)	487.0 (257–1444.5) (*n* = 9)	0.186

Values shown are n (%) or mean ± SD or median (interquartile range)

*ALT* alanine aminotransferase, *aPTT* activated partial thromboplastin time, *AST* aspartate aminotransferase, *BUN* blood urea nitrogen, *CK* creatine kinase, *CRE* creatinine. *DIC* disseminated intravascular coagulation, *Hb* hemoglobin, *PLT* platelet count, *PT* prothrombin time, *WBC* white blood cell count

**Table 2:** Laboratory characteristics of patients with *Deinagkistrodon acutus* and *Daboia siamensis* envenomation

	*Deinagkistrodon acutus* (n = 15)	*Daboia siamensis* (n = 12)	P value	OR (95% CI)
	n (%)	n (%)		
Leukocytosis (WBC > 11.0 × 10^3^/μL)	8 (53.3)	8 (66.7)	0.696	0.57 (0.12–2.75)
Moderate-to-severe thrombocytopenia (PLT < 100 × 10^3^ /μL)	12 (80.0)	2 (16.7)	0.002	20.00 (2.77–144.31)
Noncoagulation in aPTT	11 (73.3)	4 (33.3)	0.057	5.50 (1.05–28.88)
Noncoagulation in PT	13 (86.7)	7 (58.3)	0.185	4.64 (0.71–30.42)
Fibrinogen consumption (< 1 g/L)	10 (76.9)	9 (75.0)	1.000	1.11 (0.18–6.97)
Extremely high D-dimer (> 5000 ng/mL)	2 (16.7)	8 (66.7)	0.036	0.10 (0.01–0.69)
DIC score≧5	13 (86.7)	7 (58.3)	0.185	4.64 (0.71–30.42)
Acute renal impairment (CRE > 1.4 mg/dL)	3 (20.0)	5 (41.7)	0.398	0.35 (0.06–1.93)

Values shown are n (%). *N/A* not applicable

*aPTT* activated partial thromboplastin time, *AST* aspartate aminotransferase, *BUN* blood urea nitrogen, *CI* confidence interval, *CK* creatine kinase, *CRE* creatinine, *DIC* disseminated intravascular coagulation, *OR* odds ratio, *PLT* platelet count, *PT* prothrombin time, *WBC* white blood cell count

**Table 3:** Bacterial isolates identified from snakebite wounds of patients with *Deinagkistrodon acutus* envenomation who underwent surgery

Pathogen	n
Aerobic gram-positive bacteria	
Enterococcus faecalis	3
Staphylococcus aureus	2
Aerobic gram-negative bacteria	
Morganella morganii	3
Pseudomonas aeruginosa	2
Citrobacter freundii	1
Anaerobic bacteria	
Bacteroides fragilis	2

More than one type of bacteria were isolated from snakebite wound in 3 patients who underwent surgery due to *Deinagkistrodon acutus* envenomation

As to laboratory findings, both *D. acutus* and *D. siamensis* envenomation showed a certain degree of coagulopathy including thrombocytopenia, PT and aPTT prolongation, fibrinogen consumption, and elevated D-dimer levels (Table 1). However, significantly lower platelet (*P* = 0.001) and D-dimer levels (*P* = 0.008), but higher PT (*P* = 0.025) and DIC scores (*P* = 0.036) were found in patients with *D. acutus* envenomation (Table 1). A significantly higher proportion of patients with *D. acutus* envenomation presented with moderate-to-severe thrombocytopenia (*P* = 0.002; OR = 20.0, 95% confidence interval [CI], 2.77–144.31) compared to patients with *D. siamensis* envenomation. However, a significantly lower proportion of patients with *D. acutus* envenomation presented with extremely high D-dimer levels compared to patients with *D. siamensis* envenomation (P = 0.036; OR = 0.1, 95% CI, 0.01–0.69) (Table 2). The two groups did not differ significantly in WBCs, hemoglobin, fibrinogen, liver or renal function tests, or in creatine kinase levels (Tables 1 and 2).

### Features distinguishing between *Deinagkistrodon acutus* and *Daboia siamensis* envenomation

**Fig. 1:**
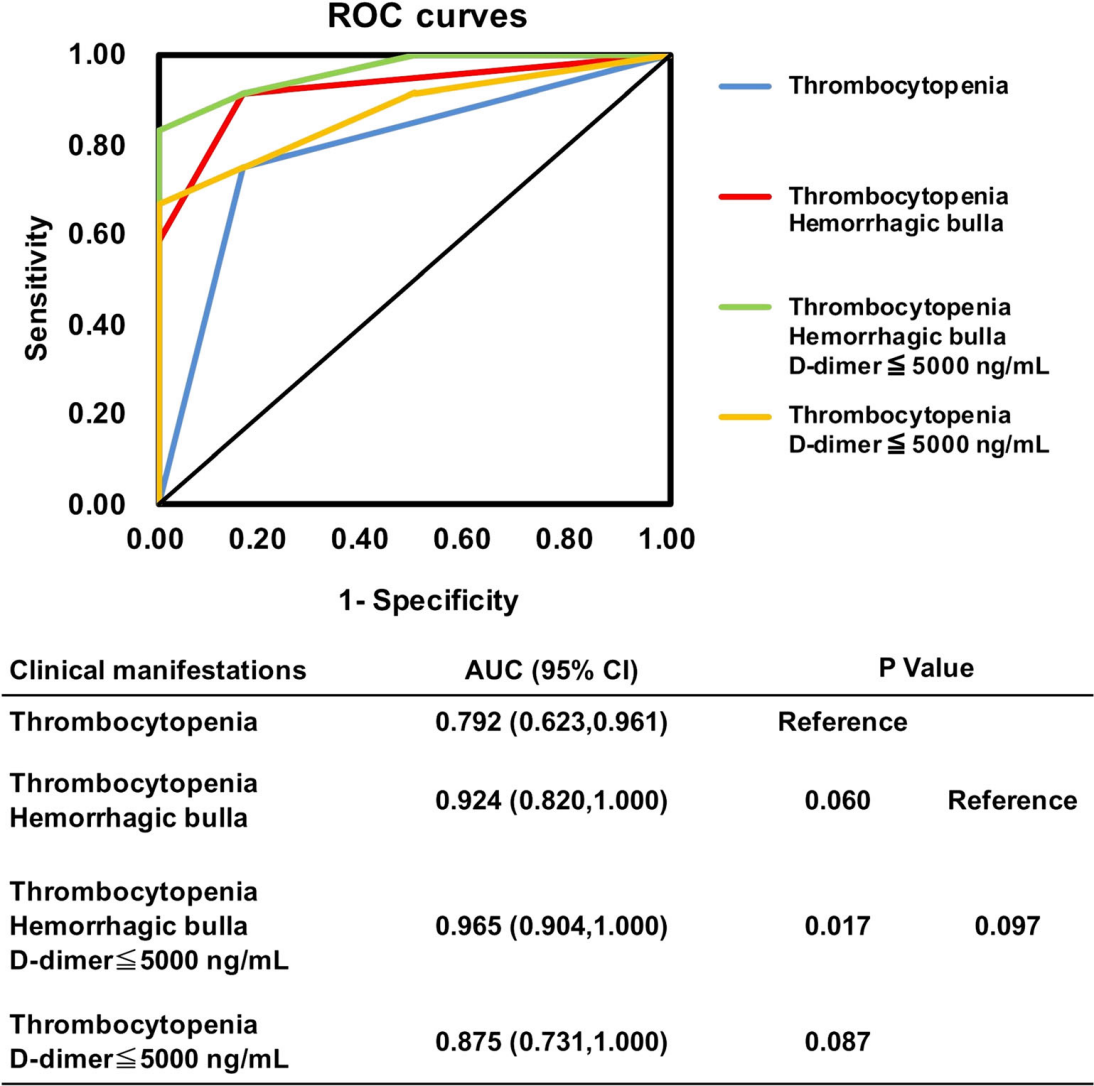
Receiver-operating characteristic (ROC) curves for the different combinations of clinical manifestations in distinguishing *D. acutus* envenomation from *D. siamensis* envenomation. Thrombocytopenia means platelet count < 100 × 10^3^/uL

We next measured and compared the discriminatory power of different combinations of clinical and laboratory features in distinguishing between *D. acutus* and *D. siamensis* envenomation by analyzing ROC curves (Fig. 1). The results showed that the combination of thrombocytopenia, hemorrhagic bulla formation, and D-dimer levels ≤5000 ng/mL had the best discriminatory power. The AUC of this combined model was significantly higher than thrombocytopenia-alone (AUC = 0.965 [95% CI, 0.904–1.00] vs. 0.792 [95% CI, 0.623–0.961], *P* = 0.017). Moreover, the presentation of both thrombocytopenia and hemorrhagic bulla was also a more apt predictor for *D. acutus* envenomation (AUC = 0.924 [95% CI, 0.820–1.00]; *P* = 0.06, when compared to thrombocytopenia-alone; *P* = 0.097, when compared to the combined model of thrombocytopenia, hemorrhagic bulla and low D-dimer levels) (Fig. 1).

## Discussion

In this retrospective study, we discovered that the presentation of hemorrhagic bulla formation and the type of surgical intervention are clinical features indicating *D. acutus* envenomation. The laboratory findings of moderate-to-severe thrombocytopenia may indicate *D. acutus* envenomation while cases of patients with extremely high D-dimer levels most likely resulted from *D. siamensis* envenomation. Combining the clinical manifestations of thrombocytopenia, hemorrhagic bulla formation and D-dimer levels can help us to distinguish between these two snakebite types.

Different from the other two venomous snakes of the Viperidae family, Taiwan habu and Taiwan bamboo viper, severe systemic coagulopathy with a DIC score **≧** 5 can be found in most instances of *D. acutus* (86.7%) and *D. siamensis* (58.3%) envenomation (Table 2). This proportion is far higher than that of the Taiwan habu (< 1%) and Taiwan bamboo viper (0%) envenomation in previous observation [6]. Moreover, noncoagulation in PT and aPTT, and severe fibrinogen consumption was also found in a significant proportion of patients with *D. acutus* and *D. siamensis* envenomation (Table 2). These findings were also uncommon in Taiwan habu or Taiwan bamboo viper envenomation [5, 6]. Thus, the above manifestation of coagulopathy may be a suitable indicator to discriminate *D. acutus* and *D. siamensis* from Taiwan habu and Taiwan bamboo viper envenomation*.*

Among the significant differences in clinical manifestation between *D. acutus* and *D. siamensis* envenomation found in our study are abnormalities in the coagulation profiles. Both *D. acutus* and *D. siamensis* venoms are composed of several hemotoxins with varying degrees of procoagulant and anticoagulant effects, which act on different steps of the clotting pathway and consume different clotting factors.

*D. acutus* venom clinically presents as anticoagulant toxins, platelet aggregation inhibitors, hemorrhagins, and TLEs [10, 12, 26, 27]. The anticoagulant toxins of *D. acutus* directly inhibit coagulation factors V and IX/X, prothrombin and tissue factors, resulting in immediate and marked prolongation of the coagulation time after envenomation [10, 28, 29]. TLEs can break down fibrinogen, but unlike real thrombin, which can activate factor XIII to perform fibrin cross-linking and stabilize fibrin clots, TLE does not form fibrin clots and produce fibrin degradation products (D-dimer) [30–33]. However, in *D. siamensis* venom the main components are phospholipase A2 and pro-coagulation proteins, which include factor V, IX, and X activators, and are very potent [14, 15, 30–32, 34]. The activators can persistently activate the coagulation pathway, and, finally, consume massive downstream coagulation factors, resulting in clotting factor deficiency, hypofibrinogenemia, fibrinolysis, and greatly elevated D-dimer levels [8, 35]. Because the main etiology leading to coagulopathy in *D. siamensis* envenomation is consumptive coagulopathy, the prolongation of coagulation time is time-dependent; severe prolongation of PT and aPTT may occur subsequently after consuming the clotting factors. The abovementioned mechanism may explain the extremely high D-dimer levels in our *D. siamensis* envenomation patients, but relatively lower D-dimer levels in *D. acutus* envenomation, as well as the finding that more *D. acutus* envenomation patients presented with non-coagulation in PT and aPTT.

Another difference found between *D. acutus* and *D. siamensis* envenomation is local wound complication. More than half of *D. acutus* envenomation patients developed extensive hemorrhagic bulla formations, requiring surgical intervention due to suspected compartment syndrome or tissue infection and necrosis. However, none of the *D. siamensis* envenomation patients presented any significant local tissue injury and none required surgical intervention. We further found that *D. acutus* envenomation patients who had undergone surgery showed evidence of wound infection. The isolated bacteria from the surgical wounds are usually a mixed spectrum, including aerobic Gram-positive and -negative, and anaerobic bacteria. This finding indicates that wound infection may partly contribute to complication in cases of *D. acutus* envenomations. The use of broad-spectrum antibiotics to cover mixed bacterial infection may be necessary in *D. acutus* envenomation. In addition, being the most venomous snake of the crotalinae subfamily in Taiwan, *D. acutus* can inject 3, 5, and 15 times the amount of venom per envenomation than that of *Protobothrops mucrosquamatus, D. siamensis* and *Trimeresurus stejnegeri,* respectively [13]. Local hemotoxic venom can affect coagulation, destroy endothelial cells and tissue, increase vascular permeability, and cause extensive vascular damage, which may explain the extensive wound complications observed in *D. acutus* envenomations [36]. Conversely, the venom amount in each injection of *D. siamensis* bite was much less than that of *D. acutus* [13]. Although, similar toxic proteins exist in the venom of *D. siamensis*, relatively weaker local tissue effects were associated with *D. siamensis* envenomation in this study; similar findings in relation to *D. siamensis* in Taiwan have been reported [8, 9].

In our study, moderate-to-severe thrombocytopenia was the most significant feature that could discriminate between *D. acutus* and *D. siamensis* envenomation. Both envenomations are likely to develop thrombocytopenia, but *D. acutus* causes more severe thrombocytopenia. Previous studies have demonstrated that *D. acutus* venom contains components that target platelets [25, 37–40]. However, the mechanism of severe thrombocytopenia in *D. acutus* envenomation has scarcely been investigated in in vitro. Nonetheless, previous studies in rat models and humans both demonstrate this result [7, 15]. In human cases of *D. acutus* envenomation, severe thrombocytopenia was found in patients within 4 h after a snakebite [7]. In rat experiments, severe thrombocytopenia was found within 10 min after injecting agkicetin-C, a potent antagonist of platelet glycoprotein Ib-IX-V, purified from *D. acutus* venom [39]. In addition to the possible direct venom effects that result in platelet consumption, sequestration of platelets by extensive tissue and vascular injury, and the severe wound infections found in *D. acutus* envenomation may all contribute to severe thrombocytopenia in humans with *D. acutus* envenomation.

However, thrombocytopenia in *D. siamensis* is thought to be related to thrombin-induced platelet aggregation and activation [34]. The pro-coagulation proteins found in *D. siamensis* venom produce massive fibrin clots and consume platelets to form systemic microthrombi [9, 41]. Although severe thrombocytopenia may also occur in *D. siamensis* envenomation, it usually takes more than 12 h after the snakebite for thrombocytopenia to occur in these patients [8].

In order to apply our findings to the clinical practice of EDs, we focused our analysis on simply defined abnormalities of coagulation profiles, such as extremely high D-dimer levels and noncoagulation in PT or aPTT, but we did not measure the optimal cutoff point via the ROC curve from individual laboratory data. Moreover, when considering that a single clinical feature may not be acceptable to accurately differentiate between these two types of snakebites, we combined different clinical and laboratory features to optimize the ROC curve. The combined model using thrombocytopenia, hemorrhagic bulla formation, and a lack of extremely high D-dimer levels had the best discriminatory power in distinguishing *D. acutus* from *D. siamensis* envenomation (AUC = 0.965 [95% CI, 0.904–1.000]). Combining the two features of thrombocytopenia and hemorrhagic bulla formation is also an acceptable diagnostic marker in distinguishing these two types of snakebites (AUC = 0.924 [95% CI, 0.820–1.000]).

There are several limitations in our study. First, this is a 13-year retrospective study and all patient data were collected from patient charts or electronic medical records. Non-uniform descriptions of signs or symptoms recorded by different physicians may influence and induce some bias. Second, although this is the largest native study regarding *D. acutus* and *D. siamensis* envenomation over the last 20 years in Taiwan, the sample size was still small due to the rarity of both types of snakebites. Third, due to the lack of a definitive guideline to manage these two snakebites in Taiwan, different treatment strategies in clinical practice might influence clinical outcomes and result in the missing values of some laboratory tests. Fourth, although we tried our best to discriminate snake species according to patient’s identification, clinical symptoms or defined criteria, there is still probable misidentification owing to non-visible real snake in ED. A prospective study conducted to validate our findings should be considered. In addition, the time-dependent change in coagulation profiles, the quantification of specific clotting factors, such as factor X, and development of the severity grading system should be considered in further studies.

## Conclusions

Among the 6 most common venomous snakes in Taiwan, life-threatening coagulopathy is frequently attributed to either *D. acutus* or *D. siamensis* envenomation. In the clinical differential diagnosis between these two types of snakebites, the presence of hemorrhagic bulla and moderate-to-severe thrombocytopenia are clinical features uniquely associated with *D. acutus* envenomation. However, extremely high D-dimer levels are indicative of *D. siamensis* envenomation.

## Abbreviations

APTT: Activated partial thromboplastin time
AUC: Area under curve
*D. acutus*: *Deinagkistrodon acutus*
*D. siamensis*: *Daboia siamensis*
DIC: Disseminated intravascular coagulation
ED: Emergency department
ORs: Odds ratios
PT: Prothrombin time (PT)
ROC: Receiver operating characteristic
TLE: Thrombin-like enzyme
WBC: White blood cell count
